# Impostorism in American medical students during early clinical training: gender differences and intercorrelating factors

**DOI:** 10.5116/ijme.5e99.7aa2

**Published:** 2020-04-29

**Authors:** Beth Levant, Jennifer A. Villwock, Ann M. Manzardo

**Affiliations:** 1 Department of Pharmacology, Toxicology, and Therapeutics, University of Kansas Medical Center, Kansas City, KS, USA; 2 Department of Otolaryngology, University of Kansas Medical Center, Kansas City, KS, USA; 3 Department of Psychiatry and Behavioral Sciences, University of Kansas Medical Center, Kansas City, KS, USA

**Keywords:** Impostor phenomenon, stress, burnout, gender, USMLE Step 1

## Abstract

**Objectives:**

This study examined the incidence and severity of impostorism in third-year medical students as they transitioned from the preclinical to clinical phases of training.

**Methods:**

A cross-sectional study was conducted in third-year medical students (N=215). Respondents completed a voluntary, anonymous, 60-item survey that included the Clance Impostor Phenomenon Scale and the Perceived Stress Scale.  Student’s-t, Mann-Whitney, and Chi-Square tests and Pearson correlation were used to determine differences between subgroups of students and relationships between instruments scores and demographic parameters.

**Results:**

Fifty-nine percent of students responded with N=112 (59% female) completing at least one instrument. The mean impostor score was 63.0 ± 14.6 (moderate-to-frequent impostor feelings) and was 9% higher in females (U=1181, p = .046). Perceived Stress scores for females were 17% higher than males (t_(109)_=2.87, p=.005).  Females had lower United States Medical Licensing Examination (USMLE) Step 1 scores (t_(107)_= 3.06,  p=.003). Impostor and perceived stress scores were correlated for males (r_(46)_=.47, p=.002) and females (r_(64)_=.54,p<.0001). Impostor and USMLE Step 1 scores were negatively correlated for males (r_(45)_ =-.32, p= .034) but not females (r_(63)_ = -.11, p=.40).

**Conclusions:**

These findings demonstrate the intercorrelation between impostorism and stress in male and female medical students and raise interesting questions regarding the contributions of gender and other factors involved with medical training.

## Introduction

Impostor phenomenon is the experience of doubting one’s accomplishments and abilities despite evidence to the contrary and fearing exposure as an “impostor”.^1^^,^^2^   The phenomenon was first described in women and believed to be a static trait, but it has since been shown to also affect men and to be a situational affective response.^3^  It is associated with psychological characteristics such as perfectionism, neuroticism, and anxiety, and can contribute to stress, loss of motivation, and the inability to enjoy successes.  Consequences of impostorism included less assertive behavior, decreased job satisfaction, and lower self-acceptance.^2^^,^^4^^,^^5^

Impostorism affects nearly half of female and one-fourth of male medical students.^6^^-^^9^ Medical students with impostorism have been found to have higher levels of psychological distress that contributes to anxiety, depression, burnout, contemplating dropping out of medical school, and suicidal ideation.^6^^,^^7^^,^^10^^,^^11^  Impostorism also impedes students’ identity formation as physicians.^12^  This detrimental effect on wellbeing can, in turn, negatively impact patient care.^13^^,^^14^

Impostor feelings are most likely to occur during periods of transition, such as beginning a career or moving between phases of a career.^15^^,^^16^  The transition from the preclinical to the clinical phases of training can be a particularly challenging for medical students,^17^^-^^19^ and is thus a time when students would be likely to experience more intense impostor feelings.  Previous studies show that students’ personality characteristics change over the course of medical education, and confidence was notably decreased by the middle of the third year of medical school.^20^^-^^23^  The proportion of medical students meeting the criterion for impostor phenomenon at a medical school in the United States of America (USA) was also found to increase in fourth year medical students, presumably related to the impending match for residency.^7^

The purpose of this study was to examine the incidence and severity of impostorism specifically in third-year medical students as they experienced the stressful early phases of their clinical training.  Differences between subgroups of students were assessed.  Relationships between instrument scores and demographic parameters were also determined to identify attributes potentially contributing to impostorism.  Noteworthy gender differences and intercorrelations were observed and are reported here.

## Methods

### Participants

This study was conducted under the authority of the University of Kansas Medical Center Office of Research Compliance who reviewed the study protocol and monitored study activities to ensure that appropriate steps were taken to protect the rights and welfare of humans participating as research participants (STUDY #00142155). Informed consent was obtained from all participants prior to participation.  Anonymous, private surveys were used to minimize the risks of participation.  The investigators did not serve as instructors for the students being surveyed.  The choice to participate was voluntary and had no impact on the students’ standing in their educational program.  Data was accessible only to the research team.  The participation incentive was designed so that no individual benefitted personally from participating.  There were no physical risks to the participants.  Psychological risks were minimal and consisted of awareness of symptoms of impostorism, stress, and burnout.  Participants were instructed that they were not required to complete any items that made them feel uncomfortable.

Third-year medical students (N = 215) of the University of Kansas School of Medicine class of 2020 participated in this study.  The University of Kansas School of Medicine had a traditional four-year program in which students underwent two years of preclinical training, consisting of a lecture-based integrated basic science curriculum with some clinical skills experiences, followed by two years of clinical training.  Students take the United States Medical Licensing Examination (USMLE) Step 1, which covers the foundational sciences, at the end of Year 2 and must pass the exam before beginning their clinical training.  Clinical training began with six required eight-week rotations to which the students were randomly assigned.  Students were located on three campuses in Kansas City, Wichita, and Salina (65%, 35%, and 0% of respondents, respectively).  Most students completed all four years on the same campus; however, a subset of students (19% of respondents) completed the preclinical phase in Kansas City and then moved to Wichita for the clinical phase.

### Data collection

A voluntary, anonymous, 60-item survey was administered in October-November of the 2018 Fall semester. This time window for data collection was selected so that students would be in the early phase of their clinical training but would have completed at least one clinical rotation.

Data were collected and managed by the authors using REDCap (Research Electronic Data Capture) tools^24^ hosted at the University of Kansas Medical Center.  REDCap is a secure, web-based software platform designed to support data capture for research studies, providing 1) an intuitive interface for validated data capture; 2) audit trails for tracking data manipulation and export procedures; 3) automated export procedures for seamless data downloads to common statistical packages; 4) procedures for data integration and interoperability with external sources. REDCap was programmed to send an e-mail to all third-year medical students containing a link to the on-line survey.

Impostorism, stress, and burnout were assessed using validated instruments.  The survey consisted of the instruments described below, as well as demographic items (e.g., age, race, gender, entering Medical College Admission Test score, medical Year 1-2 grade point averages (GPA), etc.).  It required roughly 20 minutes to complete.  Participation was incentivized by a contribution to the class fund if a specified response rate was achieved.

### Instruments

The Clance Impostor Phenomenon Scale^1^ (used with permission) was used to measure impostorism.  It is a 20-item survey in which responses are rated on a Likert scale from 1 to 5 for not at all true, rarely true, sometimes true, often true, or very true, respectively.  Responses to each item were added to yield a score ranging from 20 to 100 with a higher score indicating more frequent and intense impostorism feelings.  A score of under 40 indicates few impostor characteristics; 41-60, moderate impostorism; 61-80 frequent impostorism; and more than 80, intense impostorism.^1^ An individual was considered to have “impostor phenomenon” if their total score was 62 or higher.  This criterion for impostor phenomenon based on the evaluation of 64 participants assessed for impostor phenomenon by clinical interview and resulted in one false positive and no false negatives.^25^  The instrument has high internal reliability with Cronbach’s α=0.9226, 0.9625, and 0.87-0.89.^27^  Scores on the Clance Impostor Phenomenon Scale are related to, but were different from, measures of self-esteem, depression, social anxiety, and self-monitoring.^1^^,^^25^^,^^26^^,^^28^^,^^29^

Stress was measured using the Perceived Stress Scale^30^^-^^32^, a 10-item inventory utilising a 5-point Likert scale.  Respondents rated the frequency of stress-related feelings in the last month as 0 to 4 for never, almost never, sometimes, fairly often, and very often, respectively.  Responses to four positively stated items were reversed, and then scores were added to yield a total score ranging from 0 to 40.  The higher the score, the more stress the individual was experiencing.  The 10-item Perceived Stress Scale has a good reliability with Cronbach’s α=0.7833, 0.85 and 0.8231, and 0.71 and 0.8634 depending on the participants or the analysis of subfactors within the scale.  Scores on the Perceived Stress Scale have been shown to correlate with other stress measures, help-seeking behaviors, and smoking status.^33^ Normative values on the Perceived Stress Scale in 2009 were 15.52 ± 7.44 for males and 16.14 ± 7.56 for females.^35^

**Figure 1:**
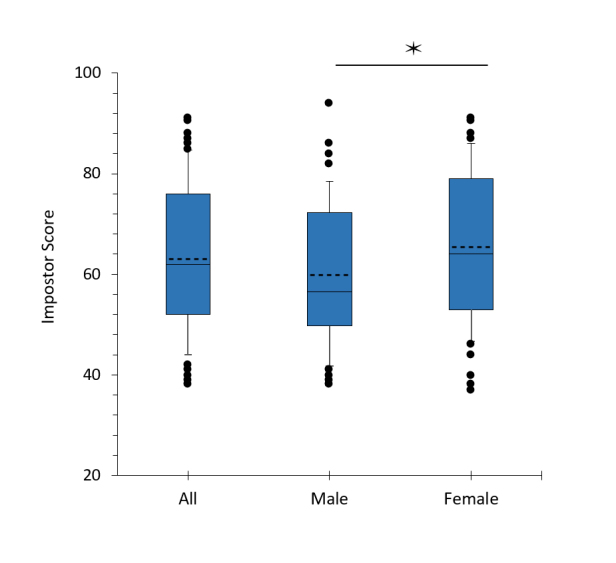
Impostor score in third-year medical students. The mean in the box plot is indicated by the dotted line. N = 112 for all students; 46 for males, 66 for females *p=.46 by Mann-Whitney test (U = 1181).

Burnout was assessed using a 2-item instrument (licensed from Mind Garden) developed for and validated in medical professionals.^36^^,^^37^ An affirmative response to either of the yes/no items was considered to indicate burnout or depersonalization, respectively. In medical professionals, responses to the single-item burnout and depersonalization questions were strongly correlated with the emotional exhaustion and depersonalization domain scores (minus the single-item questions) of the full Maslach Burnout Inventory^38^ (Spearman r=0.76-0.83 and 0.61-0.72, respectively).^36^  The predictive values of the single-item burnout and depersonalization questions were 88.2% and 89.6%, and the positive likelihood ratios were 14.9 and 23.4, respectively.^36^^,^^39^  Validation of the emotional exhaustion and depersonalization subscales of the full Maslach Burnout Inventory indicate high and moderate internal reliability (α = 0.89 and 0.67), respectively.^22^

### Data analysis

Data were analyzed using SAS statistical software (version 9.4) and Instat 3. Eight respondents omitted a response to one item on the Clance Impostor Phenomenon Scale (a different item for each respondent).  The total impostor score for these eight individuals was calculated by multiplying their score from the 19 completed items by 1.05263 and rounding to the nearest whole number.

A preliminary analysis determined no effect of campus; consequently, the campus was not used as a variable in the present analyses.  Differences in responses by gender were determined by Student’s-t, Mann-Whitney, or Chi-Square tests, as appropriate.  A non-parametric test was used for data that failed a test for normality. Relationships between scores for the individual instruments and demographic parameters were determined by Pearson correlation.

**Figure 2:**
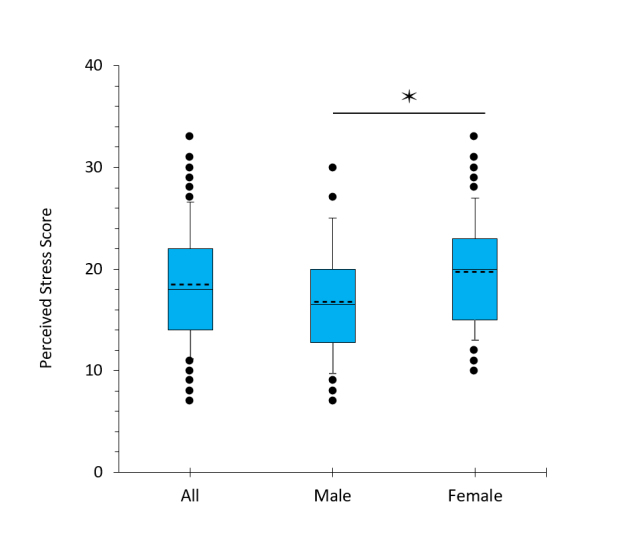
Perceived Stress score in third-year medical students. The mean is indicated by the dotted line. N = 111 for all students; 46 for males, 65 for females. *p = .005 by Student's t-test (t(109) = 2.867).

## Results

### Respondent characteristics

A total of 127 of 215 (59%) students surveyed responded with N = 112 completing at least one instrument and N= 111 completing all three instruments.  The demographic characteristics of respondents are summarized in Table 1. The respondents were generally similar to the total group except for the somewhat higher participation by females, which was not significant (χ^2^_(1) _= 2.62, p=.11).  Respondents were primarily Caucasian (83%) with a mean age of 25.8 ± 3 years.  About 8% of the sample reported Hispanic ethnicity. These individual characteristics did not significantly differ by gender.  The self-reported benchmark measures for academic performance were highly correlated.  Male and female respondents had similar grade point averages and composite examination scores.  USMLE Step 1 scores were higher in males than females (t_(107)_=3.06,  p=.003).

**Table 1:** Baseline demographic characteristics by gender

Variable	Class Mean (N%)	Respondents N	Total Mean (N%)/ Mean±SD (Range)	Male (N=46) Mean (N%)/ Mean±SD (Range)	Female (N=66) Mean (N%)/ Mean±SD (Range)	Male v. Female		
						Test^*^	df	*p-value*
Total	215	112						
Male	110 (51)			46 (41)				
Female	105 (49)				66 (59)			
Age	25.7±3	110	25.9±3 (23-44)	26.5±4 (24-44)	25.5±2 (23-34)	t = 1.56	108	*.12*
Race		112						
Caucasian	172 (80)		94 (84)	40 (87)	53 (81)	c^2 ^= 7.3	3	*.06*
African - American	7 (3)		2 (2)	1 (2)	1 (2)			
Asian	24 (11)		8 (7)	5 (11)	3 (5)			
Other	9 (4)		8 (7)	0 (0)	8 (12)			
Hispanic	13 (6)		9 (8)	4 (9)	5 (8)	c^2 ^=.036	1	*.85*
Undergraduate GPA		111	3.8±0.24 (3-4)	3.7±0.27 (3-4)	3.8±0.22 (3-4)	t = .51	109	*.62*
Composite MCAT	507±6.1	105	508±5.5 (493-525)	508±5 (500-518)	507±6 (493-525)	t = .91	104	*.36*
GPA Year 1	3.39±0.47	103	3.4±0.44 (2-4)	3.5±0.44 (2-4)	3.4±0.45 (2-4)	t = .87	101	*.39*
GPA Year 2	3.36±0.53	103	3.4±0.45 (2-4)	3.5±0.43 (2-4)	3.4±0.47 (2-4)	t = .48	101	*.63*
USMLE Step 1 Score	225±19	109	228±18 (146-264)	233±16 (146-264)	225±15 (194-256)	t = 3.06	107	*>.003*
								
Assigned Campus			Kansas City	Wichita	Salina			
Preclinical	175/32/8^**^	111	94 (84)	17 (15)	0 (0)			
Clinical	140/67/8^**^	111	71 (64)	37 (34)	0 (0)			

Data are presented as M±SD with ranges for continuous variables and frequencies with percentages for categorical variables.

^*^Statistical testing applied Student’s-t test or Chi Square test. p < .05 is considered significant. ^**^Number of students by campus:  Kansas City/Wichita/Salina

Abbreviations:  GPA – grade point average, MCAT - Medical College Admission Test, USMLE – United States Medical Licensing Examination

### Impostorism

The distribution of impostor scores is shown in Figure 1. The mean impostor score was 62 ± 15 for the entire sample and was 9% higher in females (M = 65.4 ± 14.7) than males M = 59.9 ± 14.0) (U = 1181, p = .046).   Fifty-one percent of respondents met the threshold for the impostor phenomenon (score > 62). Forty-four percent of males and 56% of females met the criterion for the impostor phenomenon, which was not different between genders (χ^2^_(1)_ =1.25, p=.26).

### Perceived stress

The overall mean perceived stress score was 18.5 ± 5.5 (Figure 2). Perceived stress scores were 17% higher for females (M = 19.7 ± 5.2) than males (M = 16.8 ± 5.5) (t_(109)_ = 2.87, p=.005).

### Relationships between variables

**Figure 3:**
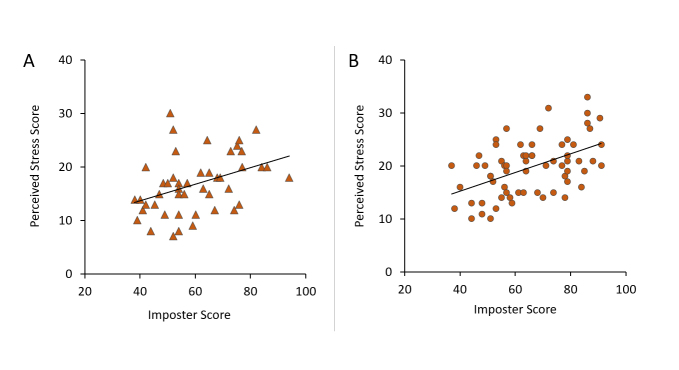
Relationship between impostor and perceived stress scores in male (A) and female (B) third-year medical students. Impostor and perceived stress scores were strongly correlated in females (r(64) =.54, p less than .001) and moderately correlated in males (r(46) = .47, p = .002) by Pearson correlation.  n = 45 for males, 65 for females.

**Figure 4:**
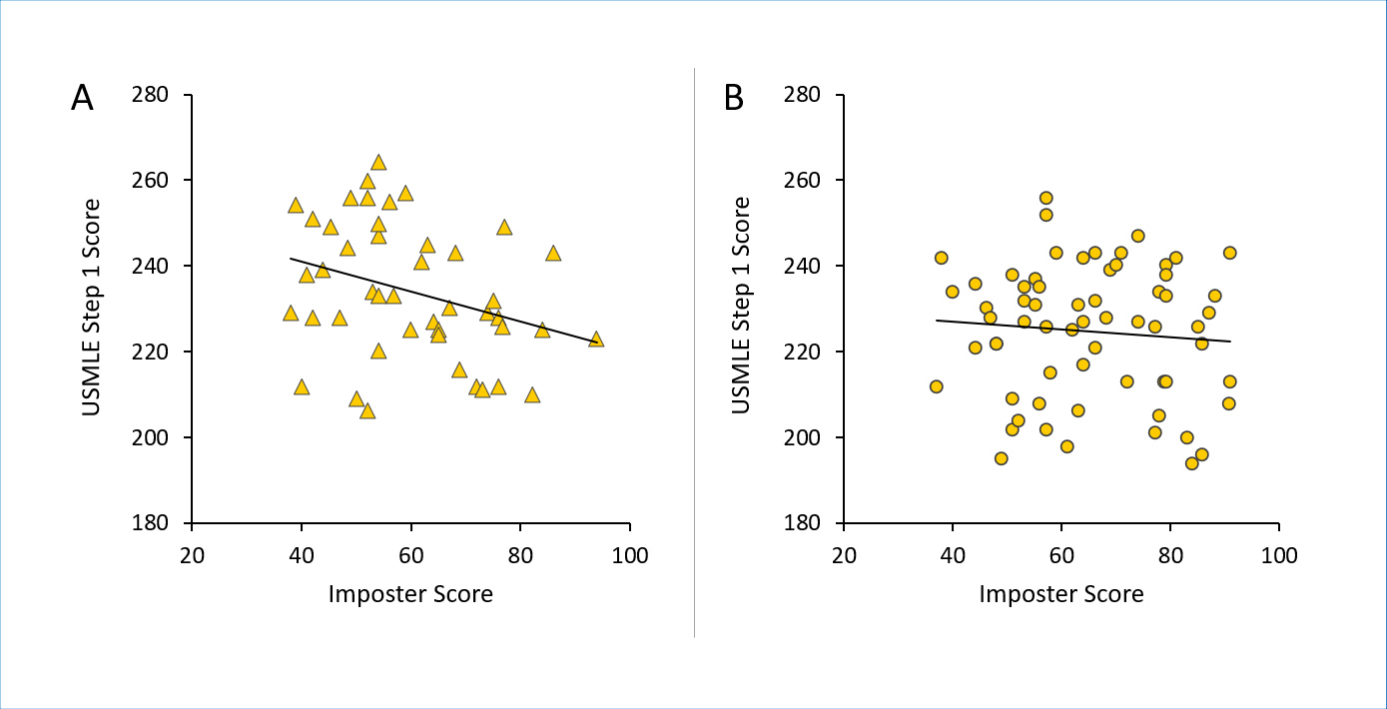
Relationship between United States Medical Licensing Examination (USMLE) Step 1 score and impostor score in male (A) and female (B) third-year medical students. A moderate negative correlation was found for impostor score and UMSLE Step 1 score in males (r(44) = -0.32, p = .034), but not in females (r(63) = -0.11, p = .40). n = 45 for males, 63 for females.

Several intercorrelations were identified between study variables that differed by gender.  Impostor and perceived stress scores were strongly correlated in females (r_(64)_ = 0.54, p < .0001) and moderately correlated in males (r_(46)_ = 0.45, p = .002) (Figure 3).  Regression lines for the relationship between impostor and perceived stress scores were roughly parallel for males (slope = 0.155) and females (slope = 0.178) indicating a similar relationship between these factors in both genders. Stress score was 28-31% higher in impostors than non-impostors for entire sample (t_(109)_=5.19, p<.0001), males (t_(44)_=2.79, p=.008), and females (t_(63)_=4.10,  p < .0001). A moderate negative correlation was also found for impostor score and UMSLE Step 1 score in males (r_(44) _= -0.33, p=.034), but not in females (r_(63)_=-0.085, p=.50) (Figure 4).  UMSLE Step 1 scores for male imposters (impostor score >62) (M = 227.6 ± 11.9) were 5% lower than non-impostors (M = 239.5 ± 16.8) (t_(43) _= 2.61 p = 0.012).

No significant relationships were identified between impostor score and age, race, undergraduate grade point average, composite Medical College Admission Test score, Years 1 and 2 grade point averages, burnout, or depersonalization for either gender (data not shown).

## Discussion

This cross-sectional assessment of impostorism in medical students examined a specific time point in medical training, encompassing the transition and early portion clinical training occurring after the completion of at least one required 8-week clinical rotation.  This is a notably stressful period and is associated with a decrease in confidence.^17^^-^^20^ The third-year medical students in this study endorsed moderate-to-strong impostor feelings (IP score >41), and 51% of students met the criterion for the impostor phenomenon. Females had slightly higher mean impostor scores than males, but the percentage of students meeting the criterion for the impostor phenomenon was not different between males and females (Figure 1). The percentage of imposters, particularly for males, was slightly higher than reported in previous studies of medical students^6^^-^^9^ and may reflect the stressors specific to the preclinical-clinical transition phase. However, this study did not assess preexisting impostor feelings at the time of admission to the medical program or whether those feelings changed across the course of medical training.

Notable relationships between study factors, as well as gender differences, were observed. First, females reported significantly more stress than males (Figure 2). This relationship is consistent with the normative values for the Perceived Stress Scale^35^ although the differences between females and males were greater in our study population than in the normative sample (17% higher v. 4% in 2009). This finding suggests core gender differences in the psychological/emotional responses to individual stressors associated with the educational process and other life experiences.  Stress was strongly correlated with the impostor score in both genders (Figure 3).

Second, females had lower USMLE Step 1 scores than males (Table 1), consistent with prior reports,^40^  even though both genders had similar indicators of academic ability and USMLE Step 1 performance, such as Medical College Admission Test scores and Year 1-2 grade point averages.^41^^-^^43^  The USMLE Step 1 exam is the first of a series of licensing exams, typically taken at the end of the preclinical phase of medical school, that has become of increasing importance as a screening tool for residency selection and is thus perceived by students as a major determinant of their professional futures.^44^  Interestingly, USMLE Step 1 score was moderately inversely correlated with impostor score only in males, despite lower levels of perceived stress in male respondents (Figure 4).  The present data do not allow elucidation of causal relationships, nor did this study assess pre-existing impostorism, which might have affected USMLE Step 1 performance. However, studies in undergraduates suggest potential underlying causes of the interrelationship between USMLE Step 1 scores and imposter feelings in the male medical students.  Test anxiety and a lack of confidence in intelligence were associated with impostorism in both males and females,^45^ which could negatively impact USMLE Step 1 performance.  However, in females, high impostorism was also associated with a higher undergraduate grade point average and time spent on academics.^5^^,^^46^  Accordingly, females with pre-existing impostorism might engage in compensatory preparation for USMLE Step 1, resulting in a higher score than might otherwise be anticipated in an individual with test anxiety or a lack of confidence, thus obscuring a relationship between impostorism and USMLE Step 1 scores.  Alternatively, poor USMLE Step 1 performance could stimulate or exacerbate impostor feelings, perhaps in concert with the effects of pre-existing impostorism.  Males with impostorism were found to react more negatively when given negative feedback than females.^47^ This suggests that negative self-appraisal of USMLE Step 1 performance could be a particularly salient factor in the male impostorism measured in this study. In contrast, the USMLE Step 1 score could have less impact on impostorism in females, perhaps due to their already higher level of impostorism.  In either case, these observations raise important questions that may inform hypothesis-driven studies of gender differences in impostorism and medical student wellness.

### Limitations

This study is limited by its cross-sectional design using self-reported data from a single class at a single medical school with a response rate of 59%.  Accordingly, the findings may not generalize to other times, schools, educational programs, or types of students.  Respondents may have different characteristics from those who declined to complete the survey.  The analyses did not control for the specific clinical rotations completed by each student.  Finally, causal relationships cannot be established from this observational study design.

## Conclusions

These findings demonstrate significant impostorism in medical students during the early phase of clinical training and indicate noteworthy differences between male and female students.  These observations raise interesting questions regarding the contributions of gender and other factors involved with medical training on academic performance and identity formation in medical students.  Future studies (e.g., longitudinal and multiple cohorts) are required to confirm the observations reported here and establish causal relationships between factors, such as the USMLE Step 1 exam, and impostorism and stress in during medical education. A greater understanding of these effects and relationships may inform efforts to foster student wellness and enhance the experience of students as they negotiate the transition from the preclinical to clinical phases of their training.

### Acknowledgements

The authors thank Drs. Giulia Bonaminio and Mark Meyer for their support and assistance in the execution of this project.  Supported by the University of Kansas School of Medicine Academy of Medical Educators and NIH CTSA Award UL1TR002366.

### Conflicts of Interest

The authors declare that they have no conflict of interest.
